# Fostering a Resilient Healthcare System: Supportive Practice Environment Initiatives to Support Nurse and Midwives a Scoping Review

**DOI:** 10.1155/jonm/8898411

**Published:** 2025-03-27

**Authors:** Gemma Doleman, Dianne Bloxsome, Rong Wang, Lisa Whitehead

**Affiliations:** ^1^School of Nursing and Midwifery, Edith Cowan University, 270 Joondalup Drive, Joondalup, Western Australia 6027, Australia; ^2^Centre for Nursing Research, Sir Charles Gairdner Osborne Park Health Care Group, Hospital Avenue, Nedlands, Western Australia 6009, Australia

**Keywords:** midwives, positive practice environment, professional development, registered nurses, scoping review, supportive, wellbeing

## Abstract

**Aim:** This scoping review aimed to explore supportive practice environment initiatives that support novice and expert nurses and midwives.

**Design:** Scoping review.

**Methods:** Studies were identified through the electronic databases Cumulative Index to Nursing and Allied Health Literature (CINAHL), MEDLINE, PsychInfo, Scopus, and Web of Science. Grey literature was searched using GreyNet, National Grey Literature Collection, ProQuest Dissertation and Theses, and Google Scholar.

**Results:** Thirty-one papers met the criteria for inclusion and data extraction. The key concepts identified were the need for adequate staffing and resources, clear and regular communication, managerial presence and engagement in committees and relationship building, celebration and recognition of staff efforts, career mapping, shared decision-making at the unit level, a positive organisational culture that promotes inclusivity and wellbeing, mentoring, support, and succession planning.

**Conclusion:** Implementation of support initiatives can increase job satisfaction for nurses and midwives across the career trajectory, resulting in them remaining in the profession.

**Summary**

• **Implication for the profession and/or patient care: Impact:** What problem did the study address? Supportive practice environment initiatives that can support nurses and midwives across the career trajectory.
• What were the main findings? Adequate staffing, effective communication, shared decision making, staff recognition, and supportive management are important for a supportive practice environment.
• Where and on whom will the research have an impact? Organisations can implement the suggested strategies in order to support and strengthen the nursing and midwifery workforce in the future.
• **No Patient or Public Contribution:** No patients or members of the public were involved in this study.
• **What does this paper contribute to the wider global clinical community?** Implementation of supportive practice strategies can improve staff wellbeing, retention, which will ultimately improve patient outcomes.

## 1. Introduction

Nurses and midwives are integral to the health and wellbeing of a country through the provision of universal health coverage, management of diseases, emergency response and preparedness and the delivery of quality and safe healthcare [1]. However, these elements are at threat of being compromised due to nurse and midwifery shortages across the globe [2]. In light of this, Buchan and colleagues [3] have urged organisations to promote positive practice environments with an emphasis on staff support, adequate staffing, and the promotion of attractive work conditions.

Magnet designated hospitals are renowned for providing positive practice environments that attract and retain nurses and midwives [4, 5]. These accredited hospitals were first described in the United States of America (USA) during the 1980 nursing shortages [6]. A magnet designated hospital is evaluated based on Magnet characteristics. These characteristics are known as the forces of magnetism and include transformational leadership, structural empowerment, new knowledge, innovation and development, excellent professional practice and outcomes [7]. Through these structures, nurses feel supported, have control over their practice [8], have adequate resources, including staffing and skill mix and are involved in hospital affairs [9, 10]. These elements result in a reduction in the rates of stress and burnout, greater levels of job satisfaction and greater retention rates [11]. Positive practice environments are also essential for positive patient outcomes and reduced adverse events [12], including rates of missed nursing care [13].

Research also focuses on the importance of managerial support for clinical nurses. Thapa et al., [14] reported that appreciation from managers is integral for nurses and midwives to feel valued in their work and acknowledged for their contribution to patient care. Cummings and colleagues [15] identified that supportive leadership resulted in positive nurse outcomes, including health and wellbeing, which promotes quality patient care. In addition, supportive leadership is considered integral for new graduate nurses and midwives entering the workforce. When support is offered to graduates, they feel valued and are less likely to leave the profession [16]. This support may include managers being available and accessible, practicing open communication, and taking a personal interest in staff [17].

Whilst research focuses heavily on the support that managers can provide to clinical nurses and midwives, there is an increase in research showing that managers also need to be supported in their roles. Currently, managers stay in their roles for a period of 2–5 years [18, 19], which costs an organisation 75%–125% of their yearly income when they leave. In addition, reduced productivity, clinical nurse/midwife turnover and higher rates of pressure ulcers are noticed when a manager leaves their position [19]. An integrated literature review identified supportive organisations as important for manager wellbeing, and when the support was lacking, manager frustration was identified. Nurse managers and supervisor relationships were also explored, with some studies reporting that managers felt that they worked in punitive environments with no relationships with their supervisors [20]. Support was also seen as integral in promoting the communication satisfaction of managers within an organisation [21].

While previous research has highlighted the importance of support in the clinical environment, no reviews have synthesised available literature on this topic from the perspective of nurses and midwives working across settings and across the career trajectory. This review will systematically identify and map the breadth of evidence available in the field and identify the key concepts in the literature related to the creation and sustainability of supportive positive practice environments.

## 2. Methods

**Figure 1:**
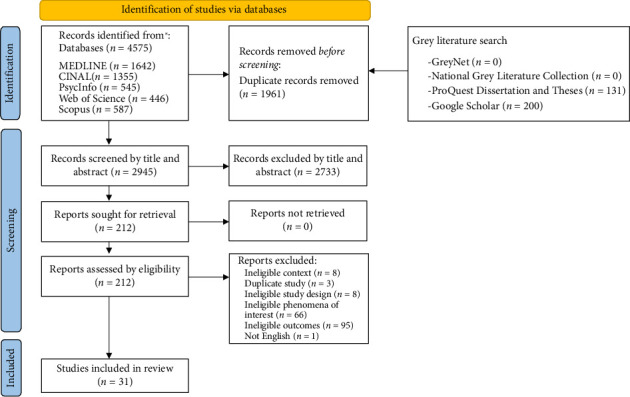
PRISMA-ScR diagram. From: Page M.J., McKenzie J.E., Bossuyt P.M., Boutron I., Hoffmann T.C., Mulrow C.D., et al. The PRISMA 2020 statement: an updated guideline for reporting systematic reviews. BMJ 2021;372:n71. doi: 10.1136/bmj.n71. For more information, visit: https://www.prisma-statement.org.

The Joanna Briggs Institute (JBI) methodology for scoping reviews was used as a framework to conduct this scoping review [22]. The results were reported using the Systematic Reviews and Meta-Analysis extension for Scoping reviews checklist (PRISMA-ScR) [23]. See Figure 1. The study was registered with Open Science Framework DOI 10.17605/OSF.IO/3PHX8.

### 2.1. Data Sources and Search Methods

Both quantitative and qualitative study designs and grey literature were considered for inclusion in this scoping review. To identify a search strategy an initial limited search of MEDLINE and CINAHL was undertaken. Following the initial search and creation of the search strategy for each database, a thorough search was undertaken. The following databases were used MEDLINE, CINAHL, PsychInfo, Scopus and Web of Science. Sources of unpublished studies/grey literature included GreyNet, National Grey Literature Collection, ProQuest Dissertation and Theses, and Google Scholar (screen 1–200 results). In addition, forward citation and handsearching was also undertaken. Studies were included if they explored supportive positive practice environment creation and sustainability for registered nurses and midwives from beginner to expert working in the hospital setting. The areas of focus included professional development, emotional and psychological wellbeing, and practical applications. The search was restricted to documents published between 2013 and 2023, written in the English language and with full text available. Excluded from the study were documents published in a language other than English.

## 3. Eligibility Criteria

### 3.1. Participants

Participants included registered nurses and midwives from novice to expert. Excluded were nurses and midwives working in the community, primary care setting, and long-term residential homes.

### 3.2. Concept

The concept includes supportive positive practice environment creation and sustainability.

#### 3.2.1. Context

The study focused on the hospital context.

#### 3.2.2. Type of Studies

This scoping review considered both quantitative and qualitative study designs for inclusion.

### 3.3. Search Outcomes

A total of 4575 studies were identified by searching the databases with an additional 331 being identified through grey literature searching. Records were searched for duplicates, and a total of 1961 studies were removed. The 2945 remaining studies were uploaded into JBI System for the Unified Management, Assessment, and Review of Information (JBI SUMARI), for screening by title and abstract. 2733 studies were excluded during abstract and title review, resulting in 212 studies for full text review. Reasons for the exclusion of sources of evidence at full text that did not meet the inclusion criteria were recorded and reported in the review. A total of thirty-one studies met the inclusion criteria and underwent data extraction.

### 3.4. Data Extraction and Synthesis

Two independent reviewers extracted data from the included papers (GD, RW). The data extracted from the included studies were specific details about the participants, concept, context, study methods and key findings relevant to the review question, see Appendix 1. A narrative synthesis was used to present the study findings.

## 4. Results

Thirty-one studies met the criteria for inclusion and data extraction. All included studies were conducted in the hospital. Of the 31 articles, 15 were conducted in the USA [24–38], five in Canada [39–43], four in Brazil [44–47], two in Turkey [48, 49], one in Jordan [50], one in New Zealand [51], one in Finland [52], one in South Africa [53] and one in Sweden [54].

Of the 31 studies, three used a cross-sectional design [38, 45, 50], one used a longitudinal design [37], three used qualitative case study design [29, 44, 46], one used a quasi-experimental design [49], two used a quantitative descriptive design [42, 53], two used a qualitative design with survey method [25, 52], five used mixed methods [26, 28, 41, 47, 51], one used non-experimental descriptive design [40], four used a qualitative design using focus groups and/or interviews [24, 36, 39, 54], two studies used a pre and post implementation survey design [30, 48], six described the implementation of a program or strategy [27, 31–35], and one used a non-predictive nonexperimental design [43].

Thirteen of the included studies explored the development or creation of a positive practice environment [25, 28, 31, 33–35, 45–48, 50, 52]. Nine studies explored leadership and or mentoring strategies [26, 29, 30, 32, 38–40, 44, 49, 51, 55] and one focused on professional development [24], four studies focused on shared governance or structural empowerment [27, 43, 53, 54], and four studies explored support strategies for graduate nurses [36, 37, 41, 42]. See supporting file for characteristics of included studies.

The key concepts identified were the need for adequate staffing and resources [33, 35, 42, 49], clear and regular communication [30, 32, 34–36, 41, 42, 44–46, 50, 53, 54], committees to support a nursing and midwifery voice [27, 33, 37, 41, 43, 47, 50, 52, 54], managerial presence and engagement with staff [25, 27, 28, 30, 34, 36, 40, 41, 45, 47, 48, 50, 54], celebration and recognition of staff efforts [28, 34, 53, 54], career mapping [29, 33], shared decision-making at the unit level [35, 53], a positive organisational culture that promotes inclusivity and wellbeing [24, 33, 36, 45], and mentoring and succession planning [25, 26, 28, 29, 31, 33, 36–39, 41, 42, 49, 52, 53]. See Table 1 for key concepts and associated definitions.

## 5. Discussion

This review aimed to explore supportive practice environment initiatives that support nurses and midwives across the career trajectory. Thirty-one studies met the criteria for inclusion and data extraction. The results highlighted the following initiatives that should be considered by management for the creation of a supportive practice environment, the need for adequate staffing and resources, clear and regular communication, committees supporting the nursing and midwifery voice, managerial presence and staff engagement, celebration and recognition of efforts, career mapping, shared decision-making at the unit level, positive organisational culture, and mentoring and succession planning.

Practice environments with favourable organisational support strengthen the workforce and positively impact on patient, nurse, and midwifery outcomes. Specifically, research has highlighted the strong correlation between the practice environment, adequate staffing, the availability of resources and job satisfaction of the nursing and midwifery workforce [56–59]. When staff are happy in their roles a reduction in turnover and burnout is identified [59, 60]. In contrast, when practice environments are not supportive through unrealistic workloads, poorly equipped facilities, unsafe working conditions, and inappropriate nurse/midwife-patient ratios, an increase in negative nurse and midwife outcomes are identified including an increase in burnout, reduced commitment to the organisation, lack of motivation and high turnover [57, 61]. In addition, patient satisfaction is significantly related to staffing shortages [62]. It is, therefore, vital that healthcare organisations establish adequate staffing and resource allocation to support nurses and midwives in their roles.

Buchan and colleagues [3]; in their report on sustainability following the COVID-19 pandemic, have highlighted the importance of creating supportive practice environments in order to retain staff and sustain the workforce into the future. This may be established and maintained through mutual respect, shared decision-making and effective organisational communication [63, 64]. Providing nurses and midwives with the platform to have their voices heard and participate in shared decision making is considered to be important for job satisfaction and retention [65]. In addition, effective, clear, and transparent communication is essential for disseminating critical information, fostering collaboration, and maintaining a cohesive work environment [66]. Effective communication, that is two ways in nature, and transparent is critical in reducing burnout [66] and reducing the use of grapevines (informal networks or information transfer) for staff to attain information not readily available to them [67]. The use of grapevines can result in demoralised workers, unrest and apathy and can contribute to an increase in turnover intentions [67]. In addition, effective, and clear communication channels also contribute to a positive organisational culture [68] and promotion of patient safety [69] and reduction in medical errors [70]. Therefore, organisations should focus on creating opportunities for nurses and midwives to be represented on committees and the formation of effective communication channels.

The importance of visible and supportive management has been well established in the literature for the promotion of job satisfaction and staff wellbeing of nurses and midwives [14, 71]. In addition, when managers are visible staff interpret this as their manager being more caring, which may also contribute to positive patient outcomes [72]. Other strategies to show manager support may include managers practicing open communication and taking a personal interest in staff [17]. Research has shown that good leadership that focuses on team collaboration and staff recognition is a protective factor against staff burnout [73, 74]. Therefore, organisations should focus on managers being visible and the recognition of staff in order to promote staff wellbeing and patient care.

This review highlights the importance of a supportive practice environment initiatives, which is particularly important in today's contemporary healthcare landscape where nursing shortages, burnout, and high turnover, to name a few, are commonly reported [75]. Scoping reviews are not designed to make practice recommendations, however, it is clear that further exploration of the link between supportive practice environments and the wellbeing of patients and nurses would be highly topical in light of the predicted 30.6 million shortage of nurses and midwives across the globe [75].

## 6. Limitations

The study is limited by selection bias as studies were only included if they were conducted in the English language and available in the databases searched. Despite these limitations, it is felt that the studies included in this review are representative of the available literature on the topic.

## 7. Conclusion

A well-designed, supportive practice environment is the foundation of an effective healthcare system. This current review highlights the importance of support, including structures and managerial support for nursing and midwifery outcomes. No review has previously synthesised the available literature on this topic from the perspective of nurses and midwives working across settings and across the career trajectory to identify the key concepts in the literature related to the creation and sustainability of supportive positive practice environments.

## Figures and Tables

**Table 1:** Key concepts and associated definitions.

Key concepts	Definition created from the literature
Adequate staffing and resources	Having adequate staff and resources available for nurses to provide adequate care. In addition, managers need to include nurses in the discussion on adequate resources and staffing to make sure that requirements are met.
Clear and regular communication	The promotion of a communication rich environment in which communications are clear, open, and honest. Communication should be shared via multiple channels including, email, bulletin boards, meetings to create a rich environment for the development of trust. This can also be facilitated by nurse managers interacting with staff to make sure that all communications are received and understood.
Committees to support a nursing voice including shared decision making at the unit level	Nurses need to have the opportunity to participate in hospital affairs through participation in shared governance committees and structural empowerment. This allows for the nursing voice to be heard at the organisational level. It is also important for nurses to be involved in shared decision making at the unit level through the creation of a team approach between managers and clinical nurses to allow for ownership of practice and expression of ideas and opinions.
Managerial presence and engagement with staff	Nurse managers need to be competent leaders that are visible on the wards, proactive, and engaged with their staff. Managers also need to mentor and coach individuals to build individual leadership capabilities for succession planning.
Mentoring and support	Managerial and peer-peer mentoring and support is needed for nurses across the career trajectory.
Celebration and recognition of staff effects	Nurses need to be recognised and rewarded for their achievements and tasks well done. The reward system should be aligned to organisational goals and objectives.
Career mapping	Career mapping and planning with nurses' is important for determining goals and future aspirations that will allow for career progression. In addition, recognising low performing staff and actively coaching them up is also important for career progression.
Positive organisational culture that promotes inclusivity and wellbeing	An environment that promotes a positive organisational culture promotes inclusivity and diversity in the nursing workforce which promotes staff engagement and job satisfaction.

## Data Availability

The data that support the findings of this study are available from online databases.

## Supporting Information

The supporting document provides a table summarizing the key characteristics of the included studies.
