# Supplementary material for: Fostering a Resilient Healthcare System: Supportive Practice Environment Initiatives to Support Nurse and Midwives a Scoping Review
Source: J Nurs Manag. 2025 Mar 27;2025:8898411. doi: 10.1155/jonm/8898411 (PMC11968167; doi:10.1155/jonm/8898411)
Supplement: Supporting Information — Additional supporting information can be found online in the Supporting Information section. [file 8898411.f1.docx]

# Appendix 1: Characteristics of Included Studies

| **Author(s)** | **Country** | **Aim of the study** | **Participants** | **Concept** | **Context** | **Study methods** | **Key findings** |
| --- | --- | --- | --- | --- | --- | --- | --- |
| **Year of** |  |  |  |  |  |  |  |
| **publication** |  |  |  |  |  |  |  |
| Al-Ruzzieh et al., | Jordan | To Identify the  differences in nurses’ perceptions of their professional practice work environment related to their participation in shared governance (SG) councils and examine the perception of effectiveness of SG councils among nurses who participate in them | n = 580 | Positive practice | Hospital | Cross-sectional | A well-defined SG council improves the perception of a positive practice environment. Nurses who participated in the shared governance council had higher scores in the subscale’s cultural sensitivity, sufficient time, staff and resources, work motivation, autonomy and control over practice and supportive leadership.  The management leadership team regularly engaged in the council work and meetings. The meetings used effective, direct, respectful communication and consensus to make decisions. |
| 2022 |  |  | nurses | work |  | design using |  |
|  |  |  |  | environment |  | questionnaires |  |
|  |  |  |  | creation |  |  |  |
| Amestoy et al., | Brazil | To analyse the strategies  used by nurses to support the insertion of dialogic leadership in the hospital environment | n = 25 nurses | Strategies to | Hospital | Qualitative Case | The strategy to support dialogic  leadership includes four key elements: dialogue, humility, setting an example, resoluteness, regular meetings, and teamwork. The need to use dialogue to support communication in the workplace and strengthen nurses' leadership was evident. Dialogic communication helped nurses strengthen the care provided. |
| 2014 |  |  |  | support dialogic |  | study |  |
|  |  |  |  | leadership |  |  |  |

| **Author(s)** | **Country** | **Aim of the study** | **Participants** | **Concept** | **Context** | **Study methods** | **Key findings** |
| --- | --- | --- | --- | --- | --- | --- | --- |
| **Year of** |  |  |  |  |  |  |  |
| **publication** |  |  |  |  |  |  |  |
| Araujo & | Brazil | To identify the kind of  work environment that should be offered by hospital leaders to their nursing staff | n = 171 | Positive practice | Hospital | Cross sectional | To stimulate positive behaviours  and attitudes among nursing staff, managers should establish clear and effective communication with their professionals to ensure role clarity, promote a good work environment, and encourage trust-based relationships. Supervisors are accessible and open to dialogue, Celebrate the success of all members, Fair distribution of bonuses and benefits, No discrimination at the workplace, Opportunity in career growth and investing in the professional development. |
| Figueiredo, 2019 |  |  | nurses | work |  | survey |  |
|  |  |  | n = 274 | environment |  |  |  |
|  |  |  | nursing | creation |  |  |  |
|  |  |  | technicians |  |  |  |  |
| Beal & Riley 2019 | USA | To describe best  organisational practices that support the development and growth of a scholarly nursing practice throughout a nurse’s full career progression | n = 32 senior | Support for | Hospital | Qualitative | The organisation's themes create  and sustain a core culture supportive of scholarly nursing practice, expectations for professional development, resources supporting scholarly  nursing practice, and senior leaders' power. Senior nurses are the drivers behind developing and creating a culture that supports scholarly nursing practice. |
|  |  |  | nurse leaders | professional |  | descriptive |  |
|  |  |  |  | development |  | research design |  |
|  |  |  |  |  |  | using interviews |  |

| **Author(s)** | **Country** | **Aim of the study** | **Participants** | **Concept** | **Context** | **Study methods** | **Key findings** |
| --- | --- | --- | --- | --- | --- | --- | --- |
| **Year of** |  |  |  |  |  |  |  |
| **publication** |  |  |  |  |  |  |  |
| Blosky & | USA | To describe how co-  workers and supervisors influence communication and workplace health; provide a mentored research opportunity for nurses without previous research experience | n = 82 nurses | Healthy work | Hospital | Qualitative | The themes of leadership actions,  shaping quality practice, and self- efficacy frame perception. Leaders must be present and pay attention to strengths, drawbacks and dimensions of power and control embedded within formal positions. Maintain interpersonal interactions with co-workers. |
| Spegman, 2015 |  |  |  | environments- |  | methods using a |  |
|  |  |  |  | Communication |  | survey |  |
| Bognar et al., | USA | To explore the  relationship between a structured leadership academy and perceived leadership practices | n = 12 nurses | Leader | Hospital | A mixed method  design using both surveys  and focus group | Common themes were leadership  roles, leadership development and relationship building. Peer-to-peer mentoring promotes collegial relationships between mentors and mentees while allowing time for a transfer of knowledge, emotional support, and sharing of advice. |
| 2021 |  |  |  | mentorship |  |  |  |
|  |  |  |  | support program |  |  |  |
| Capitulo & | USA | To describe a step-by-  step, ‘how to’ approach to the implementation of an interprofessional shared governance and relationship-based care model | Nurses | Implement | Hospital | Description of | Regularly invite council members  and managers to monthly professional governance meetings to discuss issues.  Nurse managers wrote stories about their work, including challenges, exploring, sharing, and reflecting on leadership experiences.  Nursing leadership course/workshop to advance leadership skills. |
| Olender, 2019 |  |  |  | interprofessional |  | implementation |  |
|  |  |  |  | shared |  |  |  |
|  |  |  |  | governance |  |  |  |

| **Author(s)** | **Country** | **Aim of the study** | **Participants** | **Concept** | **Context** | **Study methods** | **Key findings** |
| --- | --- | --- | --- | --- | --- | --- | --- |
| **Year of** |  |  |  |  |  |  |  |
| **publication** |  |  |  |  |  |  |  |
|  |  |  |  |  |  |  | Creating a formal infrastructure of  interprofessional councils gave staff a voice.  The shared governance and  relationships-based care model is an excellent framework for engaging and empowering staff. |
| Clausen et al., | Canada | To describe the  development of the Leadership-In-Action Program in Nursing, outlines plan for evaluation and discusses the lessons learned | 26-unit nurse | Leadership | Hospital | Group | Mentorship and coaching are  needed to support leadership development, which requires building leadership capabilities within individuals and in others. Nurse managers should be given the opportunity and support to develop competencies of the LEAP-IN program. |
| 2019 |  |  | managers | support program |  | discussion, |  |
|  |  |  |  |  |  | interviews and |  |
|  |  |  |  |  |  | focus groups. |  |
| Coelho Amestoy | Brazil | To investigate nurses’  understanding regarding leadership, as well as the strategies used for facilitating its exercising | n = 25 nurses | Positive practice | Hospital | Qualitative | Leadership is a skill that contributes  to the management of care for the team and the hospital environment. By strengthening through dialogue the main strategy of facilitating and strengthening the exercising of the nurse’s leadership. |
| et al., 2014 |  |  |  | work |  | approach using |  |
|  |  |  |  | environment |  | case study using |  |
|  |  |  |  | creation |  | workshops and  interviews. |  |

| **Author(s)** | **Country** | **Aim of the study** | **Participants** | **Concept** | **Context** | **Study methods** | **Key findings** |
| --- | --- | --- | --- | --- | --- | --- | --- |
| **Year of** |  |  |  |  |  |  |  |
| **publication** |  |  |  |  |  |  |  |
| Collins, 2016 | USA | An evidence-based improvement project to elevate nursing satisfaction rate within the ED of a large suburban hospital | n = 47 nurses | Positive practice work environment creation | Hospital | Survey, focus group | Leaders met individually with staff members to better understand their needs and desires. A reward and recognition program called “Pull up a co-worker” allows staff members to recognise each other. Post thank-you notes in the department, and those participating were eligible for gift card drawings. “Pillar” communication board in the staff break room to share service, quality, education, and people metrics.  Unit-based shared governance to include leadership presence to facilitate and offer support. |
| Colwell, 2019 | USA | To explore leadership  strategies that hospital senior nurse manager uses to improve nurse retention | n = 6 senior | Leadership | Hospital | Case study | The strategies identified included  senior nurses guiding, coaching, and mentoring registered nurses, and the retention strategies were tools to motivate and retain registered nurses. In addition, having a succession plan and assigning a leadership mentor to new nursing management leaders, Nurse manager leadership to career map nurses, work towards shared governance, professional development and transformational leadership.  Actively coach low-performing nurse up or coach them out of the department. |
|  |  |  | nurse | development |  |  |  |
|  |  |  | managers |  |  |  |  |
|  |  |  | n=3 chief |  |  |  |  |
|  |  |  | nursing |  |  |  |  |
|  |  |  | officers and 1  assistant chief nursing officer and 2 directors. |  |  |  |  |

| **Author(s)** | **Country** | **Aim of the study** | **Participants** | **Concept** | **Context** | **Study methods** | **Key findings** |
| --- | --- | --- | --- | --- | --- | --- | --- |
| **Year of** |  |  |  |  |  |  |  |
| **publication** |  |  |  |  |  |  |  |
| Connolly et al.,  2018 | New  Zealand | To examine clinical leadership of registered nurses in an emergency department | n = 37 nurses | Supports for leadership | Hosptial | Mixed method  Non-experimental survey design | ED RNs feel their ability to act as  clinical leaders is limited by a lack of structural and psychological  empowerment.  ED RNs feel they have less impact on the overall operational running of the department than on their direct clinical input. |
| Correa & Bacon, | USA | To describe the details of  leadership training program and the outcomes | 82 matched | Support program | Hospital | Pre and post | Two important concepts 1) engaging in honest and open communication serves others, and 2) leaders should engage in authentic presence. The five interventions included Work- from-home once per month, budget training, snack shack, report card created, shared, and taught to nurse managers, and personal leadership development yearly. Results  indicate that support and investment by nurse executives in the nurse manager role yield positive benefits for the Nurse managers as leaders and their staff. |
| 2019 |  |  | surveys from | for nurse leaders |  | implementation |  |
|  |  |  | nurses |  |  | survey |  |
| Dans et al., 2017 | USA | To describe the Pathway  to Excellence Program | Nurses | Framework for | Hospital | Description | Six pathway standards were  created: shared decision-making, leadership, safety, quality, culture of wellbeing, and professional development.  New nurses in the established  transition programs reported to have fewer errors, fewer negative |
|  |  |  |  | creating ideal |  |  |  |
|  |  |  |  | work |  |  |  |
|  |  |  |  | environment |  |  |  |

| **Author(s)** | **Country** | **Aim of the study** | **Participants** | **Concept** | **Context** | **Study methods** | **Key findings** |
| --- | --- | --- | --- | --- | --- | --- | --- |
| **Year of** |  |  |  |  |  |  |  |
| **publication** |  |  |  |  |  |  |  |
|  |  |  |  |  |  |  | safety practices, higher overall  competence, less stress, and more job satisfaction. |
| Good & Atchison, | USA | To describe the  Professional Practice  Development Centre  (PPDC) model | Nurses | Framework to | Hospital | Description | Providing support to our nurse  leadership team has increased the  overall efficiency of the hiring process, encouraged neutral dialogue between advisers and applicants on unit selection and has fostered professional growth within the organisation. Professional advisers schedule periodic meetings with nurse managers to provide a consistent flow of information.  The coordinator regularly  communicates with the nurse, schedules all orientation activities, and completes all requests for access resources. |
| 2022 |  |  |  | support nurses |  |  |  |
| Kol et al., 2017 | Turkey | To identify the  satisfaction levels of nurses with positive environment initiatives and positive management strategies | n = 235 | Positive practice | Hospital | Pre and post | Strategies for inclusion were  transformational leadership, structural empowerment and empirical quality outcomes. This could be achieved by Coordinators visiting all clinics for which they were responsible daily and building a close relationship with managers; two boards were created to ensure that nurses participate in both |
|  |  |  | n = 259 | work |  | intervention |  |
|  |  |  |  | environment |  | survey |  |
|  |  |  |  | creation |  |  |  |

| **Author(s)** | **Country** | **Aim of the study** | **Participants** | **Concept** | **Context** | **Study methods** | **Key findings** |
| --- | --- | --- | --- | --- | --- | --- | --- |
| **Year of** |  |  |  |  |  |  |  |
| **publication** |  |  |  |  |  |  |  |
|  |  |  |  |  |  |  | clinical and institutional decisions and increase support for the night shift from manager to coordinator level. |
| Lankshear et al., | Canada | To determine the role of  organisational power and personal influence in creating a high-quality professional’s practice environment for nurses | n = 2873 | Supports from | Hospital | A non- | The frequent use of legitimising and inspirational appeal as influence tactics may combine with organisational power to achieve the degree of manager support required to support professional practice initiatives. The study highlights the importance of organisational power and personal influence as significantly contributing to the ability of those in leadership roles to achieve desired outcomes. |
| 2013 |  |  | nurse | nurse leaders |  | experimental, |  |
|  |  |  |  |  |  | descriptive |  |
|  |  |  |  |  |  | correlational |  |
|  |  |  |  |  |  | research design |  |
| Lindfors et al., | Finland | To identify existing  orientation patterns and to find elements that may enhance successful orientation of newly graduated nurses (NGN) | n = 152 | Positive work | Hospital | Open-ended | Involve NGNs to be part of the working team, collective engagement and empowering communality create a sense of a welcoming atmosphere.  Have a systematic orientation program with adequate orientation resources to support the preceptor and NGN.  A motivated working community and a good, trusting interpersonal relationship also help create a supportive and positive  environment. |
| 2018 |  |  | preceptor of | environment |  | questionnaires |  |
|  |  |  | NGN | creation |  |  |  |
|  |  |  | orientation |  |  |  |  |

| **Author(s)** | **Country** | **Aim of the study** | **Participants** | **Concept** | **Context** | **Study methods** | **Key findings** |
| --- | --- | --- | --- | --- | --- | --- | --- |
| **Year of** |  |  |  |  |  |  |  |
| **publication** |  |  |  |  |  |  |  |
| Linnen & Rowley, | USA | Overview of recent  research and evidence about the benefits of empowerment and presents recommendations for professional nursing and healthcare leadership | Nurse | Positive work | Hospital | Description | Nurse managers or leaders should  support an inclusive unit culture that fosters open communication and shared decision-making. Create open time slots for clinical nurses and managers to connect  without interruptions. Leaders need to see themselves as stewards of the unit. Unit practice council should adopt the 5- factors of the professional practice environment model: 1) nurse advancement 2) staff participation in policy and governance, 3) nurse manager's leadership and support, staffing resource adequacy, and collegial nurses-physician relationship. |
| 2013 |  |  |  | environment |  |  |  |
|  |  |  |  | creation |  |  |  |
| Lundin et al., | Sweden | To study how first-line  managers act to make structural empowerment accessible for nursing staff, and relate these to participant’s descriptions regarding the staff’s access to empowering structures | n = 5 first-line  managers, n = 13 staff | Supports to | Hospital | Qualitative | Different channels like bulletin  boards, emails, and verbal communication were used within  the team. Having a manager present and available in the unit to provide direct support and feedback to staff improves the staff's perception of having access to structural empowerment. Staff appreciated building connections with other specialties, colleagues, and/or outside the organisation, e.g., national meetings with colleagues. |
| 2022 |  |  |  | enhance |  | descriptive |  |
|  |  |  |  | empowerment |  | design through |  |
|  |  |  |  |  |  | observation and |  |
|  |  |  |  |  |  | interview |  |

| **Author(s)** | **Country** | **Aim of the study** | **Participants** | **Concept** | **Context** | **Study methods** | **Key findings** |
| --- | --- | --- | --- | --- | --- | --- | --- |
| **Year of** |  |  |  |  |  |  |  |
| **publication** |  |  |  |  |  |  |  |
| Olmstead & | USA | To illustrate the specific  actions that the  leadership team of a hospital surgical department use to attract and retain workers | Nurses | Strategies sustain | Hospital | Description | Important concepts 1) visible  support 2) positive leadership 3)  employee appreciation 4) effective communication. Nurse leaders could achieve this by learning at least  three non-work-related facts about each employee, which enhances connection with the employee. Managers can round on the unit multiple times each shift every day. Handwritten thank you congratulations, and consolation notes to staff. Routinely recognises staff, nominates staff for awards, and publicly supports rewards and recognition. |
| Wellington, 2022 |  |  |  | a positive work  atmosphere |  |  |  |
| Oriza et al., 2016 | USA | To describe using the  healthy work  environment standards to implement a unit-based shared governance  council | Nurses | Positive practice | Hospital | Description | A shared governance council was  implemented to improve staff engagement. This was achieved through true collaboration and effective decision-making in which all nurses are responsible and accountable for the decision of the team; true collaboration and skilled communication were achieved through rounding for outcomes, unit huddles, situational awareness and  appropriate staffing through a residency program, advancement programs and meaningful and recognition of authentic leadership. |
|  |  |  | working at a | work |  |  |  |
|  |  |  | paediatric | environment |  |  |  |
|  |  |  | cardiac unit. | creation through |  |  |  |
|  |  |  |  | healthy work |  |  |  |
|  |  |  |  | environment |  |  |  |
|  |  |  |  | standards. |  |  |  |

| **Author(s)** | **Country** | **Aim of the study** | **Participants** | **Concept** | **Context** | **Study methods** | **Key findings** |
| --- | --- | --- | --- | --- | --- | --- | --- |
| **Year of** |  |  |  |  |  |  |  |
| **publication** |  |  |  |  |  |  |  |
|  |  |  |  |  |  |  | These strategies reduced turnover from 30% to 5%. |
| Pailet, 2016 | USA | To examine how chief  nursing officers (CNOs) use transformational leadership to lead and support NGNs | n = 13 CNOs | Approaches to | Hospital | Interview, | 8 major findings emerged: (a) CNOs  participate in rounds and take time to listen to new nurses, (b) CNOs create a culture of caring and compassion with new. Nurses, (c) CNOs use storytelling to create sustainable change, (d) CNOs meet with new nurses regularly, (e) CNOs recognise generational differences, (f) CNOs hire new  nurses with critical thinking and soft skills, (g) CNOs support evidence- based decision making, and (h) CNOs encourage teamwork and collaboration through shared governance. |
|  |  |  |  | support for NGNs |  | observation and |  |
|  |  |  |  |  |  | artifact reviews |  |
| Pfaff et al., 2014 | Canada | To explore the team and  organisational factors that may predict NGN engagement in collaborative practice | n = 514 new | Supports for | Hospital | Survey, | Face-to-face interactions, including  interprofessional rounds, care conferences, team meetings, and informal talk or talk to other healthcare professionals, support NGN engagement in collaborative  practice. Regular team meetings with administrators and managers to support NGN. Organisational facilitators include supportive leadership, participation in a preceptorship or mentoring experience and time. |
|  |  |  | graduate | NGNs |  | interview |  |
|  |  |  | nurse (NGN) |  |  |  |  |
|  |  |  | surveys |  |  |  |  |
|  |  |  | n= 16 NGN in |  |  |  |  |
|  |  |  | interview |  |  |  |  |

| **Author(s)** | **Country** | **Aim of the study** | **Participants** | **Concept** | **Context** | **Study methods** | **Key findings** |
| --- | --- | --- | --- | --- | --- | --- | --- |
| **Year of** |  |  |  |  |  |  |  |
| **publication** |  |  |  |  |  |  |  |
| Regan et al., 2017 | Canada | To describe graduate  nurses’ transition experience in Canadian healthcare settings by exploring the perspectives of new  graduate nurse and nurse leaders in unit level roles. | n = 42 new | Supports for NGN | Hospital | A descriptive | Both managers and graduates specified the importance of a formal orientation program, unit cultures that encourage constructive feedback and supportive mentors.  In order to achieve this NGNs need to have adequate staffing levels and manageable workloads. Mentor to support NGNs at the point-of-care for a specific period. Provide regular and constructive feedback to GNs. The New Graduate Guarantee Initiative is an Ontario-specific strategy supported by the provincial government to provide full-time job opportunities and support NGN transition in place during the transition period. |
|  |  |  | graduate |  |  | qualitative study |  |
|  |  |  | nurse (NGN), |  |  | using focus |  |
|  |  |  | n = 28 nurse |  |  | groups, |  |
|  |  |  | leaders |  |  | interview |  |
| Roji & Jooste,  2020 | South | To describe how nurse managers could support nurses in accessing structural empowerment through power resources | n = 110 | Support nurses in accessing structural empowerment | Hospital | A descriptive | Most nurses had challenges accessing structural empowerment through power sources (information, support, and resources). Necessary supplies and rewards for unusual job performance were also considered important. Nurse managers need to communicate with staff members and provide information about the status of the healthcare institution. Nurse managers provide feedback and guidance to staff members. |
|  | Africa |  | nurses |  |  | Quantitative design followed by a survey |  |

| **Author(s)** | **Country** | **Aim of the study** | **Participants** | **Concept** | **Context** | **Study methods** | **Key findings** |
| --- | --- | --- | --- | --- | --- | --- | --- |
| **Year of** |  |  |  |  |  |  |  |
| **publication** |  |  |  |  |  |  |  |
| Spector et al.,  2025 | USA | To examine the effects of NCSBN’s Transition to practice program and other transition programs in assimilating NCNs to the practice environment | n = 1088  NCNs | Transition  Program for NGN | Hospital | Longitudinal  randomised  multisite desgin | Assign a trained preceptor to guide the new nurse for the first 6 months of practice. Programs include patient-centered care, communication and teamwork, quality improvement, evidence- based practice, informatics, safety, clinical reasoning, feedback, reflection and specialty knowledge  in practice area. Encourage and support NGNs to participate in system activities during the second 6 months of the transition program, such as committees, unit projects, grandrounds, and other learning opportunities offered by the institution. |
| Torquetti et al., | Brazil | To assess the structural  empowerment of nurses  working in an emergency room | n = 21 nurses | Support in | Hospital | Survey, | Involve staff to participate in a  project at the unit level. The manager should inform the organisation’s policies and keep all staff (especially night staff) updated with the latest information and resources.  Ensure staff have adequate access to managerial support. Factors that prevent structural empowerment include partial access to support. Formal and informal power, participation in projects and insufficient material, human resources, time and organisational support. |
| 2021 |  |  | completed | working |  | interview |  |
|  |  |  | the survey | environment |  |  |  |
|  |  |  | n = 14 nurses |  |  |  |  |
|  |  |  | participated |  |  |  |  |
|  |  |  | in interviews |  |  |  |  |

| **Author(s)** | **Country** | **Aim of the study** | **Participants** | **Concept** | **Context** | **Study methods** | **Key findings** |
| --- | --- | --- | --- | --- | --- | --- | --- |
| **Year of** |  |  |  |  |  |  |  |
| **publication** |  |  |  |  |  |  |  |
| Vatan & Temel Ayla, 2016 | Turkey | To explain the effects of a formal mentoring program | n = 18 nurse leaders ( 9 mentors. 9 protegees) | Mentoring program | Hospital | Quasi-experimental | Mentor and proteges can meet and discuss the best means to establish and maintain the mentoring relationship, including common interests, goals, expectations, resources, learning styles, and personal SWOT analysis. The mentoring program positively changed leadership behaviours for both proteges and contributed towards relational job learning for mentors and personal skill development for protégées. |
| Vitale, 2019 | USA | To explore the impact of a  mentorship program on  leadership practices and job satisfaction | n = 240 | Mentoring | Hospital | Cross-sectional | Mentors and protegees need to  establish short- and long-term goals.  Protegees to outline and develop agendas for planning meetings with a mentor. An organisational liaison to provide guidance, resources, and support to both mentors and proteges. |
|  |  |  | nurses | program |  | survey |  |
| Wing et al., 2015 | Canada | To examine the  relationships between graduate nurses’ perceptions of structural empowerment, workplace incivility and mental health symptoms | n = 394 | Structural | Hospital | A predictive | Structurally empowering workplaces can lower workplace incivility and the mental health of new graduate nurses.  Nurse managers can enhance access to empowering work structure by encouraging interprofessional and interdepartmental collaboration and committee participation for  graduate nurses. |
|  |  |  | nurses | empowerment |  | non- |  |
|  |  |  |  |  |  | experimental |  |
|  |  |  |  |  |  | design |  |
